# Thyroid disease‐specific quality of life questionnaires ‐ A systematic review

**DOI:** 10.1002/edm2.357

**Published:** 2022-07-20

**Authors:** Verena Uslar, Caroline Becker, Dirk Weyhe, Navid Tabriz

**Affiliations:** ^1^ University Hospital for Visceral Surgery Carl von Ossietzky University Oldenburg Oldenburg Germany

**Keywords:** benign thyroid disease, COSMIN, endocrine orbitopathy, malignant thyroid disease

## Abstract

**Introduction:**

Thyroid diseases are very common and rarely life‐threatening. One of the main therapeutic goals is an improvement in quality of life, making it important to measure in clinical and research settings. The aim of this systematic review is to provide an overview of the currently available thyroid‐specific quality of life questionnaires with regard to their validation quality in order to make recommendations for clinical use with a special focus on German questionnaires.

**Methods:**

A systematic literature search was performed in Pubmed, Google Scholar and the Cochrane Library. A total of 904 studies were identified. After excluding duplicates, non‐English‐ or German‐language texts, full texts that were not freely available and studies with irrelevant content, 64 studies reporting on 16 different questionnaires were included in the analysis.

**Results:**

Four questionnaires concerned benign thyroid diseases (ThyPRO, ThyPRO‐39, Thy‐R‐HRQoL and Thy‐D‐QOL), six malignant thyroid diseases (THYCA‐QoL, ThyCa‐HRLQOL, EORTC‐Thy34, MADSI‐Thy, QOL‐Thyroid and ThyCAT), and six endocrine orbitopathy (GO‐QOL, GO‐QLS, TED‐QOL, STED‐QOL, TAO‐QoL and Ox‐TED). Only five questionnaires were at least developed, if not validated, in German, and five were developed in more than two languages.

**Conclusions:**

ThyPRO and the ThyPRO‐39 are the best‐evaluated questionnaires for benign thyroid diseases. Alternatively, in hypothyroid patients, the adequately validated Thy‐D‐QoL can be used. For malignant thyroid diseases, the choice should be made individually, as all six questionnaires (THYCA‐QoL, ThyCA‐HRQOL, EORTC‐Thy34, MDASI‐Thy, QOL‐Thyroid and ThyCAT) have different strengths and weaknesses. The GO‐QOL is the best‐validated questionnaire in endocrine orbitopathy. However, the TED‐QOL is also suitable as a short‐screening questionnaire for these patients.

## Graphical abstract

The aim of this systematic review was to provide an overview of currently available thyroid‐specific Qol questionnaires with focus on the quality of the validation studies and on the clinical applicability of the questionnaires and the identification of gaps regarding specific research needs. In this systematic review, a total of 16 questionnaires could be identified. For different benign thyroid diseases, the Thy‐PRO and its short version ThyPRO‐39 are well established, whereas for malignant thyroid diseases and for endocrine orbitopathy there is still some work to be done to establish a clinically usable, and rigorously validated questionnaire.

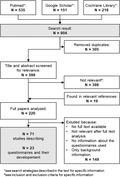

## 1 INTRODUCTION

Thyroid diseases are very common in the general population and their prevalence increase with age.^1^ Common thyroid diseases include hypo‐ and hyperthyroidism, nodular goitre, thyroid cancer and autoimmune disorders such as Hashimoto thyroiditis or graves' disease with and without endocrine orbitopathy.^2^ Due to the simple and widely available diagnostic tools, thyroid diseases are often detected early, although not every thyroid disease requires therapy.^3^

However, there is a consensus that quality of life (QoL) is negatively influenced by thyroid dysfunction, both in hyperthyroidism and hypothyroidism, and one of the main aims in the therapy of thyroid dysfunction should be at least preserving or, ideally, improving QoL.^4^, ^5^, ^6^ For this reason, the measurement of health‐related QoL has become an important issue of interest, and many instruments have been developed to measure this outcome parameter. QoL is often defined as a multidimensional subjective construct containing the dimensions of general health, physical, psychological and social functioning. It can be best measured by patients themselves in form of questionnaires using patient‐reported outcomes (PROs).^7^ Typical domains in QoL questionnaires include anxiety, impaired social life or overall quality of life. Those more general domains are usually complemented in thyroid disease‐specific questionnaires. Based on the underlying thyroid diseases these thyroid‐specific QoL questionnaires often contain domains like goitre symptoms, eye symptoms or tiredness.

Establishing relevant domains in a standardized manner should be part of the development process of each questionnaire. However, a crucial weak point of some questionnaires is this missing development step, and the lack of comprehensive assessment of measurement properties, such as validity and reliability, which prevents generalizability or comparability.^8^ Based on these shortcomings the COSMIN (COnsensus‐based Standards for the selection of health Measurement Instruments) initiative has developed a guideline for systematic reviews of PROs and a checklist for the evaluation of studies reporting on the development of PROs.^9^, ^10^, ^11^, ^12^ In 2016 a systematic review was conducted with regard to the quality of thyroid‐specific PROs.^13^ This review judges the quality of the 14 thyroid‐specific QoL questionnaires available at the time and emphasizes the need for high quality and standard reporting of the development of thyroid‐specific QoL questionnaires. However, since then, new questionnaires have been developed.

Therefore, the aim of this systematic review is on the one hand the presentation of current thyroid disease‐specific QoL questionnaires with regard to validity and reliability. On the other hand, this review focusses on the clinical usability of the respective questionnaires in order to make recommendations for clinical practice, especially with regard to validated questionnaires in the German language and in order to identify gaps regarding specific questions.

## 2 METHODS

At all times during the preparation of this Systematic Review, the PRISMA guidelines were followed.

### 2.1 Literature search

A systematic literature search has been conducted by two of the authors independently (C.B. and V.U.) in the time between 18.02.2021 and 16.03.2021. The PICO ((P) participants, (I) interventions and (C) comparators but not the outcomes) scheme was used and the databases Pubmed, the Cochrane Library and Google Scholar were searched to identify studies that assess the QoL of patients with thyroid disease through thyroid‐specific questionnaires. Initially, The Medical Subject Heading ‘quality of life’ was combined with ‘thyroid’ AND ‘questionnaire’ for a first search, and additionally combined with ‘questionnaire’ AND (‘thyroid dysfunction’ OR ‘thyroid disease’ OR ‘hyperthyroidism’ OR ‘hypothyroidism’ OR ‘graves’ disease’ OR ‘hashimoto's disease’ OR ‘thyroid cancer’ OR ‘thyroid neoplasia’ OR ‘thyroid carcinoma’ OR ‘goitre’ OR ‘thyroid autonomy’) for the second search in PubMed. Subsequently, the search was performed via the Cochrane Library and Google Scholar using the terms: ‘thyroid’, ‘quality of life’ and ‘questionnaire’. For Google Scholar, the results of the first 5 pages (50 results) were selected and supplemented by another 101 results similar to the first source of Watt et al.^14^ The type of snowballing search possible with Google Scholar may not yield the exactly reproducible results as with Pubmed or the Cochrane Library. However, as a type of add‐on like it was used here, it provides nonetheless valuable information. No time or other restrictions were applied to any search. Only English or German publications for which the full text was available were included in the search.

### 2.2 Study selection, inclusion and exclusion criteria

The process of study selection and inclusion was performed by two reviewers independently (C.B. and V.U.). In the first step, the total number of studies was reduced by excluding duplicates. Then, all titles and subsequently all abstracts were screened and checked for relevance. Relevant studies were read in their entirety. All studies for which no abstract or no complete text was available were excluded. Only studies in English or German language were included. Furthermore, only studies with human study populations were considered. In addition, those that used only general QoL questionnaires, such as the SF‐36 or the EORTC‐QLQ‐C30 questionnaire, were also excluded. Studies that used questionnaires that did not measure QoL were also not considered further. Finally, those studies that used a thyroid‐specific questionnaire but contained little information were also not analysed if there were studies about the same questionnaire presenting more information. All guidelines, reviews and meta‐analyses were excluded, but references were screened for further relevant studies. Consequently, only studies that reported on the development of a thyroid‐specific QoL questionnaire were included. After this selection process the authors C.B. and V.U. conferred about their results and any differences were resolved by consensus. The remaining studies were read and analysed by both authors independently, and relevant information was extracted, implementing an excel spreadsheet. Again, any discrepancies were resolved by consensus.

Finally, the retrieved studies were categorized into the following three groups: (1) benign thyroid disease questionnaires, (2) malignant thyroid disease questionnaires and (3) endocrine orbitopathy questionnaires.

### 2.3 Extracted information

The following information was extracted from the relevant studies if available:

- Author(s)
- Publication year
- Type of thyroid disease
- Availability in different languages
- Number of items and scales (including general content), and how they are scored
- Internal consistency and test–retest reliability, including respective values
- If, and how external validity, construct validity and criterion validity was tested
- Availability of the questionnaire
- Questionnaire processing time

## 3 RESULTS

### 3.1 Literature search

**FIGURE 1:**
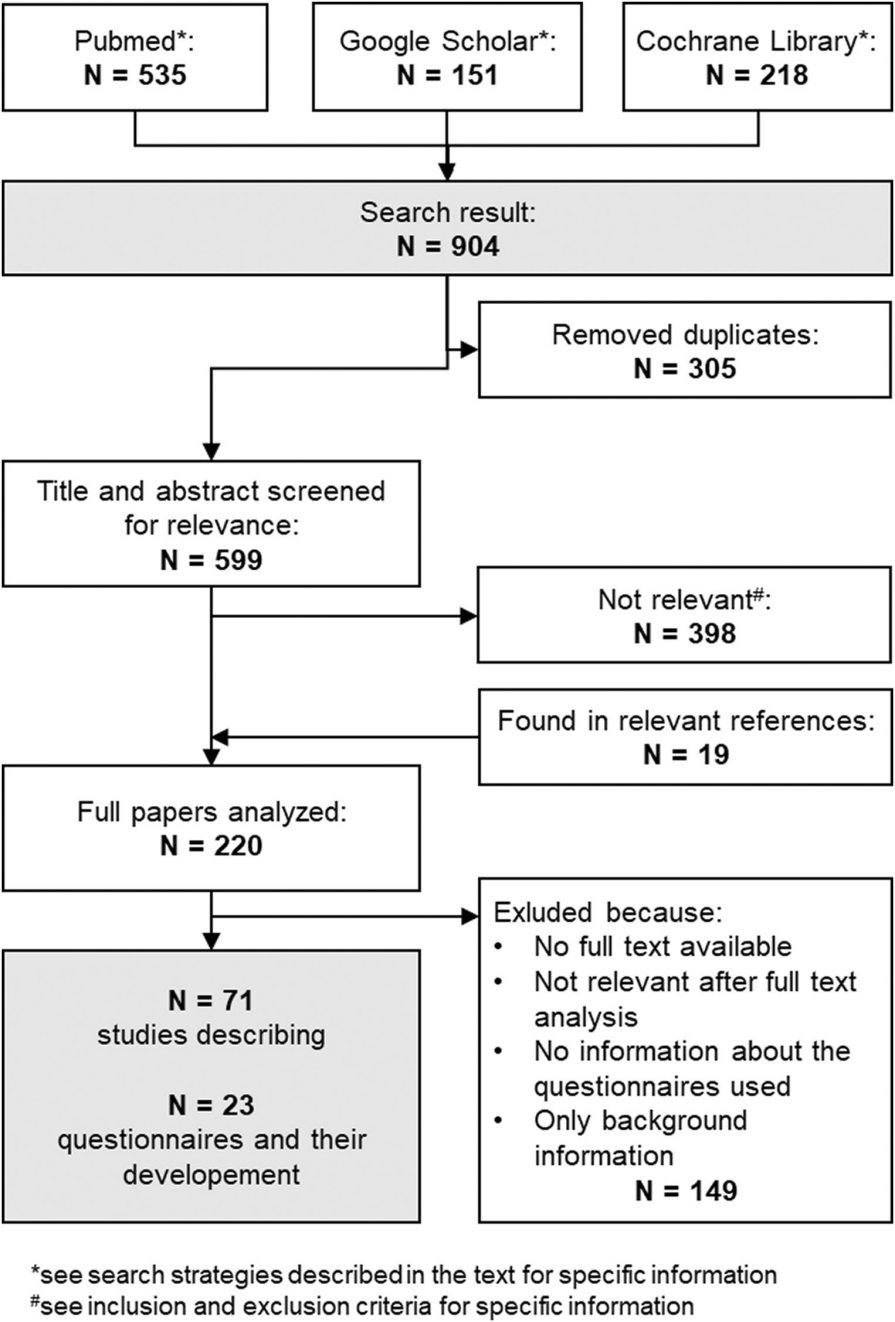
PRISMA flow chart

In Figure 1 the process of literature search, literature identification and study selection is depicted. The PubMed search yielded a total of 535 studies. The search via Google Scholar and Cochrane Library revealed a total of 369 papers. After the removal of duplicates, a total of 599 studies were screened for relevance based on their title and abstract.

This produced 220 studies, which were then read by both aforementioned authors and were independently analysed with regard to the inclusion and exclusion criteria, i.e. studies that assessed the QoL of patients with thyroid disease through thyroid‐specific questionnaires.

After further consensus, the selection was further narrowed to 71 studies, discussing the development of 16 different, relevant questionnaires. The questionnaires that did not fit the inclusion criteria were excluded because they either were more concerned with symptoms, therapy satisfaction or anxiety (ThySRQ, ThyTSQ, HCQ and WSCI‐T) or they were not specific enough (the EORTC questionnaires). Also, the NEI‐VFQ‐25 was excluded, because even from the abstract it was obvious, that the questionnaire was, albeit thoroughly validated, not useful in a research or clinical setting.

Four out of the remaining 16 questionnaires were assigned to benign thyroid diseases including hypothyroidism and Graves' disease. Six questionnaires were assigned to malignant thyroid diseases, and six questionnaires were assigned to the third category ‘endocrine orbitopathy’. In the following sections, the identified questionnaires are presented in more detail.

### 3.2 Benign thyroid disease QoL questionnaires

The following section describes the four questionnaires for benign thyroid diseases. An overview is presented in Table 1.

**TABLE 1:** Overview of the questionnaires developed for benign thyroid diseases

	ThyPRO	ThyPRO‐39	Thy‐R‐HRQoL	Thy‐D‐QOL
Authors	Watt et al.	Watt et al.	Kaniuka‐Jakubowska et al.	McMillan
Type of disease	Benign thyroid disease	Benign thyroid disease	Euthyroid goitre	Hypothyroidism
No of items	85	39	49	20
Available languages	Danish, English, Dutch, Italian, Serbian, Indian, Swedish, Spanish, Filipino, Greek, Thai, Polish, Romanian	Danish, English, Romanian, Greek, German	English, Polish	English, German, Greek, Portuguese, Turkish
Internal consistency	>0.83	‐	>0.9	0.949
Test–Retest‐Reliability	>0.77	>0.75	‐	‐
External validity	SF‐36, HADS	ThyPRO	‐	SF‐36, Beck
construct validity	Factor analysis, multitrait	Factor analysis	‐	Factor analysis
Criterion validity	Known‐groups, DIF	Known‐groups	‐	‐
Availability	✆	✆	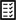	✆
Answering time	14 min	4 min	‐	_

*Note*: ‐, No information available or not evaluated.

✆: Available from author; 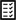
available with the following link: https://www.frontiersin.org/articles/10.3389/fendo.2018.00114/full#supplementary‐material.

#### 3.2.1 ThyPRO

The ThyPRO (Thyroid Patient Reported Outcome) questionnaire was developed and validated by Watt et al.^15^ It consists of 85 items, which are divided into a total of 13 scales and one single‐item scale. Each item has five response options on a Likert scale ranging from ‘0 = no symptoms or problems’ to ‘4 = severe symptoms or problems’ based on the period of the last 4 weeks. The higher the score, the more strongly QoL is affected.

The 13 scales include four scales on specific physical symptoms, two on mental symptoms, three on well‐being and functioning, and four on participation and social functioning. The single item asks about the impact of thyroid disease on overall QoL. Thus, different aspects of QoL are represented by the individual scales. The four symptom scales capture specific symptoms and problems in hyperthyroidism, hypothyroidism, goitre and thyroid disease with eye involvement. Thus, the ThyPRO is suitable for the assessment of QoL in all benign thyroid diseases such as Graves' disease or Hashimoto's thyroiditis.

The ThyPRO was developed based on a systemic review,^5^ interviews with patients and professionals^15^ and cognitive interviews after operationalizing the problems into items.^16^ The preliminary questionnaire was adjusted after analysing construct validity, by ‘multitrait scaling’ analysis (convergence + discriminant validity) and reliability by Cronbach's alpha.^17^ Subsequently, clinical validity was evaluated by ‘known‐groups’ and reliability by test–retest analysis of the final questionnaire.^15^ Dimensionality of the scales was confirmed by confirmatory factor analysis and the extent of differential item functioning was tested by ordinal regression.^18^, ^19^ Sensitivity to changes after clinically relevant therapies was tested and compared with the generic quality of life questionnaire SF‐36.^20^ The ThyPRO was originally developed in Danish and English but has been translated and cross‐culturally validated in many other languages such as Dutch, Indian, Italian, Serbian and Swedish.^21^ It has also been translated and validated in Greek, Filipino, Polish, Romanian, Spanish, Thai and German by other authors.^22^, ^23^, ^24^, ^25^, ^26^

#### 3.2.2 ThyPRO‐39

The ThyPRO‐39 questionnaire is the short version of the ThyPRO and has 39 items.^14^, ^27^ These are divided into 12 scales and one single item. The median response time is 4 min, which is shorter than the long version at 14 min. The development of the short version was divided into the steps of item selection, scale scoring and validation. Items were excluded for which previously missing scores were frequent, such as impaired sex life, which did not conform to item response theory, and for which differential item functioning or cross‐cultural weaknesses occurred in the previous validation studies of the long version. An additional score comprising seven scales covering mental and social well‐being and functioning was created, and the degree of agreement between the short and long scales was assessed using agreement plots and intraclass correlations.

Effect sizes and validity indices for response to the change in therapy were calculated for validation. Clinical validity was tested by the ability to discriminate between clinical patient groups. Test–retest reliability was also collected and checked with the long version. Each scale can be evaluated individually, but there is also an additional score that combines seven scales. The short version of the ThyPRO can assess QoL in many different benign thyroid diseases, and in many different languages, for instance, German, Romanian, Greek or Spanish.^22^, ^23^, ^26^, ^28^

#### 3.2.3 Thy‐R‐HRQoL

The Thy‐R‐HRQoL (Thyroid‐Related Health‐Related Quality of Life) questionnaire was created by Kaniuka‐Jakubowska et al. and is suitable for assessing QoL in euthyroid goitre.^29^ It consists of a total of 49 questions divided into 7 domains. The answer options range from ‘1 = definitely no’ to ‘6 = definitely yes’. Consequently, the QoL decreases as the score increases. In terms of content, three of the domains reflect the influence of the disease on the shape of the throat, dyspnea and performing the social role, and three others reflect the severity of subjective symptoms such as difficulty breathing, foreign body sensation in the throat, and difficulty with swallowing and hoarseness. The last domain asks about the influence of the disease on the course of comorbidities. Reliability was analysed by measuring internal consistency using Cronbach's alpha (>0.9). No further validation analyses were performed.

#### 3.2.4 Thy‐D‐QoL

The Underactive Thyroid‐Dependent Quality of Life Questionnaire (Thy‐D‐QoL) was developed by McMillan et al.^30^ It is based on the Audit of Diabetes‐Dependent Quality of Life Questionnaire (ADDQoL) and was further developed with patient interviews into a hypothyroidism‐specific questionnaire. At the beginning of the questionnaire, a question is asked about the current general QoL, which can be rated on a seven‐point scale from ‘+3 = excellent’ to ‘−3 = extremely poor’. This is followed by a question about how the QoL would be without hypothyroidism, which is answered on a scale from ‘−3 = much better’ to ‘1 = worse’. The other 18 domains refer to the influence of hypothyroidism on individual aspects of QoL, such as leisure time, work, family, social environment, energy, appearance and depression. Again, these domains ask what the problems would be like if they did not have hypothyroidism. The response options for these 18 domains are again from ‘−3 = much better’ to ‘1 = worse’. Each of the domains is then rated on how important the aspect was to the patients, from ‘3 = very important’ to ‘0 = not at all important’. The two questions in a domain can then be multiplied to give a weighted domain score, which can range from ‘−9 = maximum negative’ to ‘+3 = maximum positive’. However, the 18 domains can also be combined into an overall average weighted score. The lower the score, the worse the QoL. For internal validation, Cronbach's alpha was determined with a value of 0.949. A forced one‐factor analysis was also performed, which confirmed that all domains can be combined into an average score. The focus of this questionnaire is clearly on QoL rather than symptoms, as the authors also developed a questionnaire for symptoms of hypothyroidism only, the Thyroid Symptom Rating Questionnaire (ThySRQ), and one for the effects of L‐thyroxine therapy, the Thyroid Therapy Satisfaction Questionnaire (ThyTSQ).^31^ Quinque et al. performed a validation of the German version of McMillan's questionnaires. The German version of the Thy‐D‐QoL also has an underlying factor with factor loadings >0.4, a Cronbach's alpha of 0.92, item‐total correlation of >0.2 and low to moderate correlations with generic quality of life questionnaires such as the SF‐36.^32^

#### 3.2.5 Further questionnaires for benign thyroid diseases

In addition to the questionnaires mentioned above, further questionnaires for the assessment of QoL in benign thyroid diseases were found in the literature search. Some of these were not validated and only developed for one study specifically or only capture a partial aspect of QoL, such as physical and mental symptoms. Four studies measured QoL in subclinical and manifest hypothyroidism with nonvalidated questionnaires designed for the author's own studies.^33^, ^34^, ^35^, ^36^ Scerrino et al. compiled a questionnaire to measure QoL after total thyroidectomy in patients suffering from Graves' disease from various validated questionnaires such as the MOS‐24, the SF‐36 and the GO‐QOL. This contained 36 items covering clinical, neurovegetative, psychosocial and daily aspects, and scar quality. Except for a pilot test, which was intended to check the understanding of the items but also the test–retest reliability, no analyses of validity were performed.^4^ McMillan et al. created, in addition to the Thy‐D‐QoL, the ThyTSQ, the Thyroid Treatment Satisfaction Questionnaire, and the ThySRQ, the Thyroid Symptom Rating Questionnaire. In terms of content, the ThyTSQ can be divided into two sections. One measures current treatment satisfaction with seven items, and one measures past treatment satisfaction with four items. The ThySRQ is a list of 15 symptoms for which occurrence and severity can be rated. Both questionnaires have a high reliability of 0.9 for the ThyTSQ and 0.8 for the ThySRQ.^31^, ^37^ The Hyperthyroid Complaint Questionnaire (HCQ) was developed by Fahrenfort et al. in Dutch to quantify somatic and mental problems that occur in the context of hyperthyroidism.^38^ It contains 31 items determined by patient interviews. Individual symptoms can be scored as to whether they are ‘currently present’, ‘previously present’ or ‘not present at all’. The ‘base scale’ can be calculated from the number of all currently present symptoms and an ‘additional scale’ from the number of previously present symptoms. Item‐total correlations were >0.4 for 29 items, and reliability, as measured by Cronbach's alpha, was 0.93. The Cooper questionnaire measures the extent of six symptoms associated with hypothyroidism and whether they have worsened or improved over time.^39^ No validation is performed yet.

The literature search revealed two further questionnaires that have been used to assess QoL in thyroid diseases. They address QoL after thyroid surgery and have been used in both benign and malignant thyroid disease.^6^, ^40^ In addition, Wilde et al. developed the German‐language HPQ‐40/28 questionnaire to assess QoL in hypothyroidism.^41^ The original version consists of 40 items, and the shortened one of 28 items. Factor analysis was performed to confirm the five scales depression and anxiety, loss of vitality, pain and cramps, neurovegetative symptoms and gastrointestinal symptoms. The reliability of these scales measured by Cronbach's alpha ranged from 0.74 to 0.88.

Lastly, Jaeschke et al. investigated the frequency of importance of patients' symptoms attributed to hypothyroidism.^42^ They established a wide variety of patient‐specific complaints associated with hypothyroidism, which might be helpful to investigators interested in the development of thyroid‐specific QoL questionnaires.

### 3.3 Malignant thyroid disease QoL questionnaires

The following section describes the six questionnaires for malignant thyroid diseases. An overview is presented in Table 2.

**TABLE 2:** Questionnaires for malignant thyroid diseases

	THYCA‐QoL	ThyCa‐HRLQOL	EORTC‐Thy34	MADSI‐Thy	QOL‐Thyroid	ThyCAT
Authors	Husson et al.	Li et al.	Singers et al.	Gning et al.	Ferrell, Dow et al.	Aschebrook‐Kilfoy et al.
No of items	24	22	34	25	30	10
Available languages	Dutch, Korean	English, Filipino	See EORTC website	English	English, Korean	English
Internal Consistency	0.45–0.82	0.73–0.87	‐	0.76–0.92	0.74–0.94	‐
Test–retest reliability	‐	‐	‐	‐	‐	‐
External validity	EORTC	EORTC	‐	‐	FACT‐G (0.78)	NATCSS
Construct validity	Factor analysis, multitrait	Factor analysis, Divergence, convergence	‐	‐	‐	Consistency with bifactor model
Criterion validity	‐	‐	‐	ECOG	‐	‐
Availability	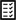	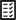	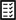	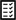	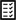	‐
Answering time	‐	‐	15 min	‐	‐	< 2 min

*Note*: ‐: No information available or not evaluated.

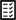
: available in the publication (ThyCa‐HRQOL (45), MDASI‐Thy (48)) or with the following links: THYCA‐QoL (https://informahealthcare.com/doi/full/10.3109/0284186X.2012.718445), EORTC‐Thy34 (https://www.eortc.org/app/uploads/sites/2/2018/08/Specimen‐THY34‐English.pdf), QOL‐TV (https://www.midss.org/sites/default/files/qol‐thy.pdf).

#### 3.3.1 THYCA‐QoL

The THYCA‐QoL (Thyroid Cancer‐Specific Quality of Life) questionnaire was developed by Husson et al. as an add‐on to a general QoL questionnaire, the EORTC‐QLQ C30, to assess specific aspects of thyroid cancer patients.^43^ The THYCA‐QoL consists of 24 items covering patient's complaints for the past 4 weeks, which are assigned to seven scales. These scales cover neuromuscular symptoms, voice, concentration, local symptomatic symptoms, throat and mouth, psychological and sensory complaints. The response options are similar to those of the general EORTC questionnaire. Thus, there are four response categories ranging from ‘1 = not at all’ to ‘4 = very much’. The evaluation also follows the scheme of the EORTC. An increasing score reflects a decreasing QoL.

In the first phase of the development, a literature review, focus group discussions and an evaluation of the relevance of the identified problems by experts and patients were conducted. In phase two, the final problem list was operationalized into questions. Finally, in phase three, first a pretest with a debriefing and then a second pretest to check the scales structure was performed. This was confirmed by factor analysis. The Cronbach's alpha values range from 0.45 to 0.82, which means that only four of the seven scales are above the limit (>0.7) for reliability. The THYCA‐QoL was originally developed in Dutch and English and translated and validated in Korean by Jeong et al.^44^

#### 3.3.2 ThyCa‐HRLQOL

The Thyroid Cancer‐Specific Health‐Related Quality of Life Questionnaire (ThyCa‐HRLQOL) was developed by Li et al. in Filipino and English.^45^ It contains 22 items, which are assigned to five scales. Thematically, the five scales include perceived anxiety, psychological distress, functionality, voice and throat complaints. The focus here is more on psychological and functional complaints, compared with the THYCA‐QoL, which focuses on symptoms. This questionnaire was also developed as a specific extension for thyroid cancer patients to the general EORTC‐QLQ C30 questionnaire.^46^ Thus, the response period, the response options and also the scoring are the same as for the EORTC and THYCA‐QoL Questionnaire.

The questionnaire is based on a literature review, focus group discussions and an assessment of the problems for relevance by patients and experts. Initially, a preliminary version was constructed as a pilot test. Subsequently, the resulting questionnaire has been analysed with respect to scaling, validation and reliability. Construct validity was tested by factor analysis, convergence and divergence validity by the Spearman correlation and the five‐factor scale structure by a scree test. The results of the analysis indicate good validity and the Cronbach's alpha values of 0.73–0.87 depending on the scale indicate good reliability.

#### 3.3.3 EORTC‐Thy34

The EORTC‐Thy34 (Thyroid Cancer Module of the EORTC) is developed by Singer et al.^47^ The questionnaire contains 34 items divided into 10 scale items: discomfort in the head and neck, fatigue, anxiety, hair problems, restlessness, influence on job or education, swallowing, worry about other things, tingling or numbness, voice and into six individual items: body image, cramps, dry mouth, temperature intolerance, joint pain, shoulder problems. The response options range on a four‐point scale from ‘1 = not at all’ to ‘4 = very much’, which refer to the past week and can be transformed into a score from 0 to 100. A high score correlates with a high symptom burden and is associated with low QoL. Since the beginning, patients from different regions of the world have been involved in the process of questionnaire development. As a result, it is already available in 15 languages: Arabic, Chinese, Dutch, English, French, German, Greek, Hebrew, Hindi, Japanese, Italian, Polish, Portuguese, Spanish and Tamil.

The questionnaire was based on two literature reviews and structured interviews with patients and professionals. The problems identified were rated by them for relevance and importance and shortened to a list of 47 items. This provisional item list was tested and evaluated with debriefing interviews. Criteria previously established according to EORTC guidelines determined which items were retained, modified or excluded. In addition, items were categorized into hypothetical scales, and Cronbach's alpha and item‐scale correlations were evaluated. Psychometric testing to validate the final questionnaire is still to be conducted in the fourth phase of development.

#### 3.3.4 MDASI‐Thy

The MDASI‐Thy (Thyroid Cancer Module of the M. D. Anderson Symptom Inventory) was developed as a supplement to the MDASI, a questionnaire that measures symptoms and its impact on activities of daily living in carcinoma patients, by Gning et al. for thyroid cancer patients.^48^ The MDASI‐Thy is composed of six items with specific symptoms such as hoarseness, feeling hot, feeling cold, palpitations, difficulty in swallowing and diarrhoea, 13 items on general cancer symptoms from the original MDASI and six interference items that capture the impact of the disease on activities of daily living in the past 24 h. These include general activity, emotional state, influence on work and relationships with other people, walking and enjoyment of life.

Each item can be scored on an 11‐point scale ranging from ‘0 = nonexistent/no influence’ to ‘10 = as bad as you can get/totally bothers you’. Various scores can be calculated by averaging the scores of the included items. A ‘symptom severity score’ can be formed from the 19 symptom items, a thyroid sub‐score from the six thyroid‐specific items, with a larger score indicating a higher symptom burden and impact on daily life.

The thyroid module was created based on a literature review, focus group discussions with patients and experts, additional interviews and a ‘cognitive debriefing’ after completing the questionnaire. Reliability, measured by Cronbach's alpha, ranged from 0.76 for the Thyroid Scale to 0.92 for the Interference Scale. Furthermore, ‘known‐groups’ validity was demonstrated, as the MDASI‐Thy can be used to distinguish different patient groups.

#### 3.3.5 QOL‐Thyroid

The Quality of Life ‐ Thyroid (QOL‐Thyroid) questionnaire was developed by Ferrell et al. and contains 30 items.^49^, ^50^ These are assigned to four scales, each reflecting an aspect of QoL. Physical well‐being is measured with two items and 13 questions, psychological well‐being with 13 items and 22 questions, social concerns with eight items and 14 questions, and spiritual well‐being with seven items and questions. Response options range from ‘0 = worst outcome’ to ‘10 = best outcome’ on an ordinal 11‐point scale, with items 1, 3, 10–23 and 27 scored inversely. This should also be considered in the evaluation when calculating an average score or a scale score.

The thyroid version of the questionnaire is based on a general questionnaire for assessing the QoL of patients with cancer, which was also developed by Ferrell et al. and the City of Hope Institute. This was based on a literature review, patient interviews and a pilot test. Psychometric evaluation of validity and reliability, with factor analysis, correlations to the validated FACT‐G questionnaire,^51^ Cronbach's alpha and test–retest analysis showed good results.

Results on the validation of a previous thyroid version, which consisted of 41 items, were described by Dow et al.^52^ Cronbach's alpha values ranged from 0.74 to 0.94 on the scales and 0.97 overall. A moderate to high correlation of 0.78 was found with the FACT‐G questionnaire. A translation of the English version into Korean was performed by Ryu et al. with subsequent validation.^53^ For this, item convergence and discriminant validity were measured by Pearson correlation, internal consistency by Cronbach's alpha, test–retest reliability by intraclass correlation and external validity by Spearman correlation of an item with the VHI‐30 questionnaire.

#### 3.3.6 ThyCAT

The special feature of ThyCAT is that it is not a classical questionnaire but a computer adaptive test to measure the QoL of patients suffering from thyroid cancer. It was developed in English by Aschebrook‐Kilfoy et al.^54^ The questions are taken from the questionnaire of the NATCSS, North American Thyroid Cancer Survivorship Study. The NATCSS questionnaire includes the City of Hope Quality of Life Thyroid Version questionnaire by Ferrell et al., and other open‐ended QoL questions, and includes a total of 75 items.^55^ The ThyCAT consists of 58 of these questions. Each participant answers different items. Depending on how the first question is answered, a provisional score is formed. This decides which question will be asked next to get the most information. Using a linear regression model, it was confirmed that the ThyCAT scores predicted the QoL scores of the entire NATCSS questionnaire with a precision of 0.96. Validity was demonstrated by the degree of agreement of ThyCAT with the bifactor model on which it is based.

#### 3.3.7 Further questionnaires for thyroid cancer

The questionnaire of Emmanouilidis et al. has a focus on QoL after thyroidectomy and radio ablation therapy.^56^ Pak et al. focus on symptoms and complaints associated with radio ablation therapy and hypothyroidism.^57^ The Hebrew questionnaire created by Dagan et al. (TQOLI, Thyroid Quality of Life Instrument) consists of a total of 15 items that were composed of items from the University of Washington Quality of Life Instrument for Head and Neck Tumours and the City of Hope Quality of Life Thyroid Version by Ferrell et al. described earlier.^58^

### 3.4 QoL questionnaires for endocrine orbitopathy

The following section describes the six questionnaires for endocrine orbitopathy. An overview is presented in Table 3.

**TABLE 3:** Questionnaires developed for endocrine orbitopathy

	GO‐QOL	GO‐QLS	TED‐QOL	STED‐QOL	TAO‐QoL	Ox‐TED
Authors	Terwee et al.	Yeatts et al.	Fayers et al.	Wong et al.	Tehrani et al.	Insull et al.
No of items	16	9	3	10	90	7
Available languages	See EUGOGO website	English	English, Korean	Chinese	German	English
Internal consistency	0.89 V 0.97 A	0.89	‐	‐	0.63	‐
Test–retest reliability	0.83 V, 0.87 A	_	0.74 V, 0.87 A, 0.81 LQ	‐	‐	‐
External validity	MOS‐24, SIP	NEI‐VFQ 25	GO‐QOL, GO‐QLS	_	‐	‐
Construct validity	Factor analysis	Factor analysis	‐	Factor analysis, Rasch analysis	‐	‐
Criterion validity	✓	✓	✓	✓	(✓)	‐
Availability	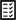	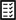	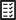	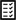	✆	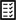
Answering time	3,1 min	2,4 min	1,6 min	‐	‐	‐

*Note*: ‐: No information available or not evaluated.

✓: Correlation with clinical parameters (NOSPECS classification, CAS or Herthel index).

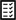
: available in the document (GO‐QOL (59), GO‐QLS (65), TED‐QOL (66), STED‐QOL https://tvst.arvojournals.org/article.aspx?articleid=2705521, Ox‐TED (69)).

✆: Inquiries to the author.

#### 3.4.1 GO‐QOL

The original Graves Ophthalmopathy Quality of Life Questionnaire (GO‐QOL) was developed in Dutch by Terwee et al.^59^ With a total of 16 items, it measures ‘visual functioning’ on the one hand (eight items) and psychosocial effects of the altered appearance on the other hand (also eight items). The visual functioning scale can be answered on a three‐point scale with ‘not limited, a little limited, strongly limited’ and the appearance scale with ‘strongly, a little, not at all’, each referring to the past week. For scoring, the eight questions in each case are summed and transformed into a scale from 0 to 100, where 0 represents the worst health status and 100 the best. To create the GO‐QOL, relevant items from existing vision‐specific quality of life questionnaires such as the VF‐14, ADVS and VR‐SIP were used, based on discussions from patients and experts. In addition, a questionnaire with open‐ended questions related to symptoms and limitations due to the disease was completed by patients and subsequently analysed. A pretest was conducted before using the GO‐QOL. The two‐scale structure was confirmed by factor analysis. Validity has been demonstrated by correlations with individual scales of other questionnaires and with age, sex and clinical measures. Both scales show good reliability as measured by Cronbach's alpha values of 0.89 and 0.87. This was also confirmed by test–retest analysis, with intraclass correlations of 0.83 and 0.87 (59). Further analyses measured good long‐term validity and found that clinical change was well‐captured by the GO‐QOL.^60^, ^61^

Over time, modified versions of the questionnaire have been published in Croatian, Danish, Dutch, English, French, German, Greek, Italian, Russian, Spanish and Turkish. It was also adapted for the Australian population.^62^

#### 3.4.2 GO‐QLS

The Graves' Ophthalmopathy Quality of Life Scale (GO‐QLS) was developed by Yeatts et al.^63^ A total of nine items include restrictions on social functioning, ‘distance activities’ such as recognizing faces in a room or watching a movie, ‘near activities’ such as finding something on a crowded shelf and restrictions on general vision such as the appearance of the eyes and the influence of symptoms on well‐being. The two general items can be answered on a five‐point scale, the remaining items on a six‐point scale. A high score represents a more impaired QoL.

In a first step, a 105‐item questionnaire was assembled from SF‐12, DSQL, NEI‐VFQ‐51 and disease‐specific questions on visual acuity.^64^ For the GO‐QLS, only those items were selected that had matching response scales, few missing values, correlated best with disease severity and discriminated best between mild and moderate disease severity. Unidimensionality was demonstrated by factor analysis. The validity of the final GO‐QLS was tested by correlation with the NEI‐VFQ‐25 and clinical disease severity scores. The Cronbach's alpha value was 0.89.

#### 3.4.3 TED‐QOL

The TED‐QOL (Thyroid eye disease Quality of Life Questionnaire) developed by Fayers et al. is the shortest with a total of three items.^65^ The items enquire about the influence of endocrine orbitopathy on general QoL, on daily activities and on satisfaction with the own appearance. The extent of the influence can be evaluated on an 11 point scale, where 0 stands for ‘no influence’ and 10 for ‘totally disturbing’. Each item is evaluated with a separate score. There is no composite score for all three items. The values of each item can be linearly transformed to a score of ‘0 = worst possible outcome’ and ‘100 = best possible outcome’. Interviews with experts and patients and a pilot test were performed. A high positive correlation of the scales of the TED‐QOL with the corresponding scales of the GO‐QOL and the GO‐QLS questionnaires indicates good validity. Correlation with clinical scores such as the VISA score was also tested. Test–retest reliability was demonstrated by intraclass correlation coefficients ranging from 0.74 to 0.87.

#### 3.4.4 STED‐QoL

The STED‐QoL (Singapore Thyroid Eye Disease Quality of Life Questionnaire) includes 10 items and was developed by Wong et al. specifically for Chinese patients with endocrine orbitopathy.^66^ The items represent four aspects of QoL with the domains activity limitations, comfort, psychological and social. The questions are answered on a four‐point Likert scale from ‘0 = all the time’ to ‘4 = not at all’. Consequently, a high score represents a good QoL. A total of six psychosocial items can be evaluated separately in a subscale. Qualitative analyses of focus group discussions were conducted to create the STED‐QoL. The resulting questionnaire was pilot‐tested and modified according to psychometric analyses. For validation, a factor analysis was performed to confirm unidimensionality, and a Rasch analysis was performed to test the ‘item response theory’. Furthermore, the ability of the questionnaire to discriminate between different disease activities was tested using ANOVA analysis. Evaluations of reliability have not yet been performed.

#### 3.4.5 TAO‐QoL

The TAO‐QoL (Thyroid Associated Ophthalmology Quality of Life) questionnaire was developed by Tehrani et al.^67^ The aim was to develop a questionnaire for German patients with endocrine orbitopathy, which measures the QoL also after surgical therapy. It contains 90 items and was designed in collaboration with ophthalmologists and endocrinologists. Four categories ranging from ‘1 = maximum satisfaction’ to ‘4 = minimum satisfaction’ are available for response. From all answers, the average can be calculated for a total score, where low values indicate a high QoL. The validation was indirectly demonstrated by a correlation between the QoL scores and clinical parameters (Hertel value). However, a correlation value was not available. Reliability, measured by Cronbach's alpha, was low with a value of 0.63.

#### 3.4.6 Ox‐TED quality of life score

The Ox‐TED (Oxford Thyroid Eye Disease) Quality of Life Score was created by Insull et al.^68^ The questionnaire consists of seven questions about the influence of the disease, the therapy, the changed appearance on the general QoL and on daily activities. The questions can each be answered with a scale from ‘1 = does not bother’ to ‘10 = very bothersome’. All scores were added together for evaluation. The total score ranges from 7 to 100. The greater this score, the greater the influence on QoL. Information on validity and reliability is not available.

#### 3.4.7 Further questionnaires for endocrine orbitopathy

Two more questionnaires could be identified that did not have validity and reliability testing. Finamor et al. created a 10‐item questionnaire called GO‐HRQL with three response categories (‘0 = not impaired, 0.5 = somewhat impaired and 1 = very impaired’).^69^ The score ranges from ‘0 = minimal’ to ‘10 = maximum’ impact on QoL. It includes psychosocial aspects such as change in appearance and influence on self‐esteem and social contacts, and visual function aspects such as walking or reading. The second questionnaire was developed by Sisson et al.^70^ It consists of four items with the four response categories ‘0 = none to 3 = strong’, which ask for the pain in the eyes, changed appearance and visual acuity.

## 4 DISCUSSION

The aim of this systematic review was to provide an overview of currently available thyroid‐specific Qol questionnaires with a focus on the quality of the studies by considering the validation and reliability process. In contrast to the work of Wong et al.,^13^ the focus is not so much on the assessment of the quality of the questionnaires, as this is described clearly and in detail there. Instead, the focus is more on the clinical applicability of the questionnaires and the identification of gaps regarding specific questions.

In this systematic review, a total of 16 questionnaires could be identified, and others that capture only partial aspects of QoL. Since the assessment of Qol depends on the underlying thyroid disease, the questionnaires are divided into three main groups.

For benign thyroid disease, four specific questionnaires were identified. Of these, three have been evaluated in sufficient quality to be used for studies. These include the ThyPRO, the ThyPRO‐39 and the Thy‐D‐QoL.^14^, ^15^, ^30^ The ThyPRO questionnaire is well‐validated and has high reliability for assessing the QoL of benign thyroid diseases and should be used for questions examining QoL as the main outcome. A comparison between the different benign thyroid diseases can also be made through its different subscales. A disadvantage of the ThyPRO is its length of 85 items and long completion time. This could result in incomplete patients' responses. The ThyPRO‐39 is much shorter and faster to answer, which is certainly an advantage over the long version. Another advantage of the short version is that items that previously showed weaknesses in cross‐cultural validation or differential item functions were excluded. The validity of the Thy‐D‐QoL by McMillan et al. was evaluated in fewer steps compared with both versions of the ThyPRO, but it still shows good quality. The Thy‐D‐QoL could be used in research with hypothyroidism as a focus or in the clinical setting, as it is even shorter than the ThyPRO‐39. For the goitre‐specific questionnaire by Kaniuka‐Jakubowska, the Thy‐R‐HRQoL, validity has not been tested.^29^ Nevertheless, it has high reliability and could be used as an additional questionnaire for questions investigating the effects of goitre. One advantage is that with a total of 49 items it addresses possible problems caused by goitre very specifically. The Thy‐R‐HRQoL and the ThyPRO‐39 were developed after the publication by Wong et al.^13^ Thus, Wong's recommendation for the use of the ThyPRO to assess QoL in benign thyroid disease is also confirmed by this review. However, it should be noted that a validated German version of the ThyPRO‐39 questionnaire is now available, which can be used for the research on benign thyroid diseases.^28^

For malignant thyroid diseases, six Qol questionnaires were identified. Three of the questionnaires have been developed as specific supplements to the valid, general EORTC questionnaire. These include the THYCA‐QoL, the ThyCa‐HRQOL and the EORTC‐Thy34 module. Overall, none of the questionnaires was evaluated with regard to all possible evaluation aspects. However, the EORTC‐Thy34 should be a well‐evaluated tool in the foreseeable future. Another important aspect is the availability of validated translations. This is the case only in Dutch and Korean for the THYCA‐QoL and in English and Filipino for the ThyCa‐HRQOL. The EORTC Thy34 offers an advantage here, as it has been developed in 15 languages, including German. The focus of the MDASI‐Thy questionnaire is more on symptoms and their effects than on other domains of QoL. Thus, this is more suitable for questions with a corresponding focus and not for questions that want to investigate, for example, psychosocial aspects.

The only psychometric tests used in the MDASI‐Thy evaluation were the analysis of internal consistency and the ‘validity of known‐groups’. A major weakness of the City of Hope Institute QOL‐Thyroid is that the validation by Ferrell et al. was only performed for a general questionnaire, on which the specific versions for malignant thyroid disease are based. Subsequently, only Cronbach's alpha values and the correlation of a scale with the corresponding one of the FACT‐G questionnaire were checked by Dow et al.^51^, ^52^ This questionnaire was the first to be developed and has been used by many studies. The use of this questionnaire is supported by the fact that its content covers not only physical but also psychological, social and spiritual aspects. The ThyCAT is the only computer adaptive test. A big advantage is that it is very short with less than 2 min of answer time for 10 questions. Disadvantages are that it requires a smartphone or computer to access the software to complete and reliability has not been tested. In terms of content, it is based on the QOL‐Thyroid. No test–retest analysis was performed on any of the questionnaires. In addition, criterion validity was only examined for the MDASI‐Thy. Similarly, response time or completion rates are an important aspect to evaluate the questionnaires. Response times were only reported for the ThyCAT and the EORTC‐Thy34, thus a comparison with other questionnaires in this respect is not possible. In conclusion, each of the six available questionnaires has strengths but also weaknesses. A recommendation for a single questionnaire cannot be given, as individual consideration should be given to which drawbacks have the least impact on use in the planned study or the clinical setting. For a quick screening, the ThyCAT is certainly a good option. If the focus is more on specific symptoms, the MDASI‐Thy could be the right choice. If different dimensions of QoL or a healthy control group are required, the EORTC questionnaire with a specific module will be a good choice.^47^ If this module is sufficiently validated in the future, it will probably become the gold standard for assessing QoL in malignant thyroid disease research.

Out of the six specific questionnaires that were identified on the topic of QoL in endocrine orbitopathy, only three are useful for clinical and research use. The TAO‐QoL and Ox‐Ted do not have sufficient quality for use, and the STED‐QoL was developed exclusively for Asian patients. The GO‐QOL questionnaire by Terwee et al. is the most commonly used disease‐specific QoL questionnaire. The GO‐QLS, on the other hand, has a focus on the impact of impaired vision, and less on consequences due to altered appearance. In contrast to the two questionnaires, the three items of the TED‐QoL are less specific and superficial but quick to answer. Therefore, the TED‐QoL is well‐suited for a quick screening on general limitations of Qol or in studies in which Qol is measured as a secondary outcome parameter. A comprehensive analysis of aspects affecting QoL is not possible with the TED‐QoL, in contrast to the GO‐QoL and the GO‐QLS. Response times and completion rates for these three questionnaires are reported by Fayers et al.^65^ The completion times are short at 3 min or less for all three questionnaires.

The GO‐QOL is the best‐validated questionnaire. Reliability and external, construct and criterion validity are high. An advantage over the TED‐QOL and GO‐QLS is that GO‐QOL is the only questionnaire for which longitudinal validity has been confirmed. Thus, a comparison of QoL before and after therapies is possible with the GO‐QOL. Nevertheless, both the GO‐QLS and the TED‐QOL are sufficiently validated for clinical and research use. An additional advantage of the GO‐QOL is the available validated translations for various languages, which can be downloaded from the site of the European Group on Graves Orbitopathy (EUGOGO). A point of criticism is the fact that the item ‘impairment while cycling’ is not relevant for some cultures. Thus, it was replaced by Park et al. in creating an Australian version.^62^ The GO‐QLS and the TED‐QOL have not yet been translated and validated in German. Only the TAO‐QoL by Tehrani et al. is available in German but in insufficient quality.

In the course of time, several reviews have been made on the assessment of QoL in endocrine orbitopathy.^71^, ^72^, ^73^, ^74^ Since the most recent reviews by Bartalena et al. and Lee et al. in 2019 and 2020, no new recording instruments or further validations of the questionnaires listed here have been published. An approach for future research could be either the validation of German versions of the GO‐QLS and the TED‐QoL, and an improvement of the German TAO‐QoL, in order to have validated survey instruments available to assess QoL in patients with endocrine orbitopathy in clinical settings or studies with a German‐speaking population.

There are certain limitations to this review. Firstly, we did not provide a quality appraisal per se or an analysis of bias. However, as this is no typical systematic review, we did not deem it necessary. Also, the tables detailing the different questionnaires and our comments with regards to the different questionnaires in the discussion section provide enough data and information to allow the reader to make an informed decision on which questionnaire might be relevant for them. Secondly, we did not include questionnaires dealing only with the aspect of anxiety. The respective questionnaires are mentioned, but since anxiety is only one aspect influencing the multi‐faceted QoL, we deemed it beyond the scope of this review to go into detail there.

Our review indicates that currently for malignant thyroid diseases and for endocrine orbitopathy there is still some work to be done to establish a clinically usable and rigorously validated questionnaire. In case of malignant diseases, this will probably be no issue anymore in the foreseeable future, when the thyroid‐specific EORTC questionnaire is available. However, as mentioned above, the currently available questionnaires for endocrine orbitopathy are not sufficiently validated or do not cover all aspects of thyroid‐specific QoL. Thus, future research should cover this currently existing gap.

## 5 CONCLUSIONS

This review offers a guide for researchers in need of a thyroid‐specific Quality of Life questionnaire as it gives an overview of currently available questionnaires and the way they were validated, with a focus on their clinical applicability. For different benign thyroid diseases, the ThyPRO and its short version ThyPRO‐39 are the most suitable questionnaires for clinical use. In hypothyroidism, the Thy‐D‐QoL can be used as an alternative. The choice of a QoL questionnaire for malignant thyroid diseases should be decided individually. For the assessment of QoL in patients with endocrine orbitopathy, the GO‐QOL is best validated. As a short screening questionnaire in the clinic or for questions in which QoL is a secondary outcome, the TED‐QoL seems to be an appropriate alternative.

## AUTHOR CONTRIBUTIONS

**Verena Nicole Uslar:** Data curation (equal); investigation (equal); methodology (equal); supervision (equal); validation (equal); visualization (equal); writing – original draft (supporting); writing – review and editing (lead). **Caroline Becker:** Data curation (equal); formal analysis (equal); investigation (equal); visualization (equal); writing – review and editing (equal). **Dirk Weyhe:** Conceptualization (equal); project administration (supporting); resources (lead); writing – review and editing (supporting). **Navid Tabriz:** Conceptualization (lead); methodology (equal); project administration (lead); supervision (equal); validation (equal); writing – original draft (lead); writing – review and editing (equal).

## FUNDING INFORMATION

This research did not receive any specific grant from any funding agency in the public, commercial or not‐for‐profit sector.

## CONFLICT OF INTEREST

The authors have no relevant financial or nonfinancial interests to disclose.

## ETHICAL APPROVAL

The conducted research is not related to either human or animal use.
